# Supplementary material for: Systematic Study of Gold Nanoparticle Effects on the Performance and Stability of Perovskite Solar Cells
Source: Nanomaterials (Basel). 2025 Oct 1;15(19):1501. doi: 10.3390/nano15191501 (PMC12525810; doi:10.3390/nano15191501)
Supplement: Supplementary file 1 [file nanomaterials-15-01501-s001.zip › nanomaterials-3734785-supplementary.pdf]

Supplementary Materials

Systematic Study of Gold Nanoparticle Effects on the Performance and Stability of Perovskite Solar Cells

Sofia Rubtsov [1], Akshay Puravankara [1,2], Edi L. Laufer [1], Alexander Sobolev [1], Alexey Kosenko [1], Vasily Shishkov [1], Mykola Shatalov [1], Victor Danchuk [3], Michael Zinigrad [1], Albina Musin [3] and Lena Yadgarov [1,*]

[1] Department of Chemical Engineering, Biotechnology and Materials, Faculty of Engineering, Ariel University, Ariel 40700, Israel
[2] Department of Physics, Faculty of Natural Sciences, Ariel University, Ariel 40700, Israel
[3] Central European Institute of Technology—Nanotechnology, Brno University of Technology, 16200 Brno, Czech Republic
* Correspondence: lenay@ariel.ac.il

Materials and Methods:

1. Substrate cleaning protocol:

For basic cleaning, glass substrates (soda-lime 0215 Corning Glass, Corning, USA) measuring 75 × 50 × 1 mm were sonicated in a Micro-90 cleaning solution (International Products Corporation, USA) diluted 1:50 with distilled water at 80 °C for 10 minutes using an ultrasonic cleaner (GT Sonic-P3, China). The substrates were then rinsed with deionized water to remove any remaining cleaning solution, followed by ultrasonic treatment in deionized water for an additional 10 minutes. Afterward, the substrates were dried under airflow and sonicated in isopropanol at 80 °C for 10 minutes. Afterward, they were blown dry with nitrogen, immersed for 5 seconds in boiling bi-distilled water (Milli-Q® IQ 7003, Merck, Germany), dried once more in nitrogen, and finally heated in an oven (MRC MSF 11-4, Israel) at 120 °C for 20 minutes. Cleaned substrates were stored in a vacuum desiccator. Prior to use, the glass was cleaved into 25 × 25 mm squares. Each part was rinsed in a spin coater (WS-650HZ-23NPPB, Laurell Technologies Inc., USA) sequentially with 3 mL bi-distilled water, 0.5 mL isopropanol, 3 mL bi-distilled water, 0.5 mL isopropanol, and twice more with 3 mL bi-distilled water, spinning at 4000 rpm. A final spin was performed at 6000 rpm for 15 seconds. After rinsing, the substrates were dried in an oven at 120 °C for 20 minutes.

2. TiO_2 BL deposition

Right before the blocking layers deposition, the substrates were processed in a vacuum plasma cleaner (MTI Corp. EQ-PCE3, USA) for 2 min at a "High" regime in residual air at 0.18 Torr.

The TiO_2 BL was deposited on FTO substrates by VST TFSP-842 (VST, Israel) magnetron sputter. Before the deposition, the vacuum chamber was evacuated by the turbomolecular pump to a vacuum no worse than 2.2×10^{-6} Torr. The pressure of working gas (Ar) in the chamber was 10 mTorr. The deposition power (RF) applied to the TiO_2 magnetron target (Testbourne LTD, 99.99% purity, 2-inch diameter, 0.125-inch

thickness) was 100W. To ensure TiO_2 BL purity, the target was pre-sputtered for 20 minutes with the closed sample shutter under the said conditions. The deposition time was 20 minutes at room temperature. The uniformity of the coating was ensured by rotating the sample table at 10 rpm. The thickness of TiO_2 layers was defined by the gravimetric method.[1] The thickness of the samples was 60 nm.

3. BL treatment method

The TiO_2 BL annealing was carried out in a vacuum furnace at 550°C for 60 minutes under a continuous airflow of 600 ccm. This process aimed to enhance the crystallinity of the layer, reduce surface hydroxyl groups, and modify the surface morphology to improve electron transport and increase hydrophobicity. For the printed TiO_2 microdot array with embedded gold nanoparticles (TiO_2-Au_MDA), the annealing process involved slow heating in the same vacuum furnace, gradually reaching 550°C. The temperature was then maintained for 30 minutes under the same oxygen flow conditions (600 ccm). This thermal treatment facilitated both the crystallization of TiO_2 particles within the ink and the formation of gold nanoparticles while also removing residual solvents and stabilizing the printed structure.

The annealing stages are as follows: 30° → (200 min) 500° → (50 min) 550° → (60 min) 550° → (60 min) 400° → (10 min) 400° → (60 min) 300° → (10 min) 300° → (60 min) 200° → (10 min) 200 → (60 min) 100° → (40 min) 60°.

4. Materials

The TiO_2 NPs (TNP) were synthesized with a sol-gel method, using titanium isopropoxide ($Ti[OCH(CH_3)_2]_4$, 99.995%, Alfa Aesar, UK), nitric acid (HNO_3 70%, Daejung,) and isopropanol (C_3H_8O Carlo Erba). Ink with gold complex nanoparticles (AuCNP) was synthesized from hydrogen tetrachloroaurate(III) trihydrate ($HAuCl_4{\cdot}3H_2O$ 99.99%, Alfa Aesar), ethylene glycol (Daejung), and surfactant Tween 80 (Sigma-Aldrich, Germany). Bidistilled water (DW) (Milli-Q® IQ 7003, Merck, Germany) was used in all experimental procedures.

5. Preparation of TiO_2 nanoparticles

TiO_2 nanoparticles were prepared via a low-temperature peptization technique. Initially, 0.7 mL of nitric acid (HNO_3) was combined with 100 mL of deionized water and heated to 70 °C under vigorous magnetic stirring. Once the solution reached the desired temperature, 12 mL of isopropanol was introduced, followed by the gradual dropwise addition of 14 mL of titanium isopropoxide. The mixture was maintained at 80 °C with continuous stirring for 45 minutes, then covered with aluminum foil and allowed to stir at room temperature for an additional five days. The resulting colloidal TiO_2 sol was transferred to a plastic container and left to dry slowly under ambient conditions for five more days, leading to the formation of a solid phase. The obtained xerogel was collected without

undergoing calcination and stored at room temperature for further applications.[43]

6. Preparation of TiO_2-xAu ink

The ink presenting a dispersion of AuNP and TiO_2 in the mixture of EG and water was prepared with a modified polyol method on TiO_2 support.[2] The 0.125 g of TiO_2 NPs xerogel was dissolved in 6 ml EG and 2 ml DI water. The prepared colloid solution was heated to 150ºC in a flask immersed in an EG bath; vapors were cooled down in a jacketed double coil condenser (44 cm diameter) with circulating water at 15ºC and returned to the flask. The acid solution consisted of 0.0677 g, 0.1 g, and 0.134 g (0.017 M, 0.025 M, and 0.034 M) of $HAuCl_4·3H_2O$ mixed with 2 ml DI water and 1.2 µl Tween 80, which was immediately injected into the flask with boiling TiO_2 colloid. The mixture was boiled for 10 min under constant, intensive stirring (500 rpm) with a magnetic stirrer. Next, the resulting dispersion of AuCNP was cooled to room temperature and stored at 8ºC in a lab refrigerator. [43]

7. Printing methods

The MDA patterns of TiO_2 and TiO_2-AuNP were deposited on the substrates using a Dimatix Materials DMP 2850 printer (Santa Clara, CA, USA) with a 10 pL cartridge. Each dot was obtained from a single jetted droplet with a drop spacing of about 160 µm. The drop spacing was chosen to be greater than the contact diameter of a single drop on a substrate (90/40 µm) so that the drops could not merge. The printing was carried out with a jetting frequency of 5 kHz, the voltage on the nozzle was varied in the range of 19 to 25 V, and only one nozzle was used to print the pattern. During printing, the temperature of the substrates was stabilized at 30°C. After printing the patterns, the samples were annealed at 550°C for 60 min with a 60

SCCM flow of dry air (ALIMC-500 ccm, Alicat Scientific, USA) in the Tube Furnace KJT1200 (Zhengzhou Kejia Furnace Co., Ltd). The final reduction of Au and the development of

TiO_2-Au_MDA was followed during the annealing of the samples.[6]

8. Solar Cell Device Structure

High absorbance and low interfacial resistance are critical for optimizing the PSC in the solar cell fabrication process. Herein, an increased absorbance is achieved by adding TiO_2Au_MDA. At the same time, low interfacial resistance is attained by the microdot architecture of the additional layers. Here, we fabricate three types of PSCs of a planar architecture: reference devices with a structure presented in Scheme S1A, modified PSCs with TiO_2_Au_MDA on the BL/MAPI interface, Scheme S1B, and a diode-like structure with TiO_2Au_MDA on the BL/HTL interface - Scheme S1C. The energy level diagram of PCS with TiO_2-Au_MDA is presented in Fig. 1SI. The chosen MDA architecture of the additional layers promotes MAPI percolation, thus reducing the effective resistivity. [3]

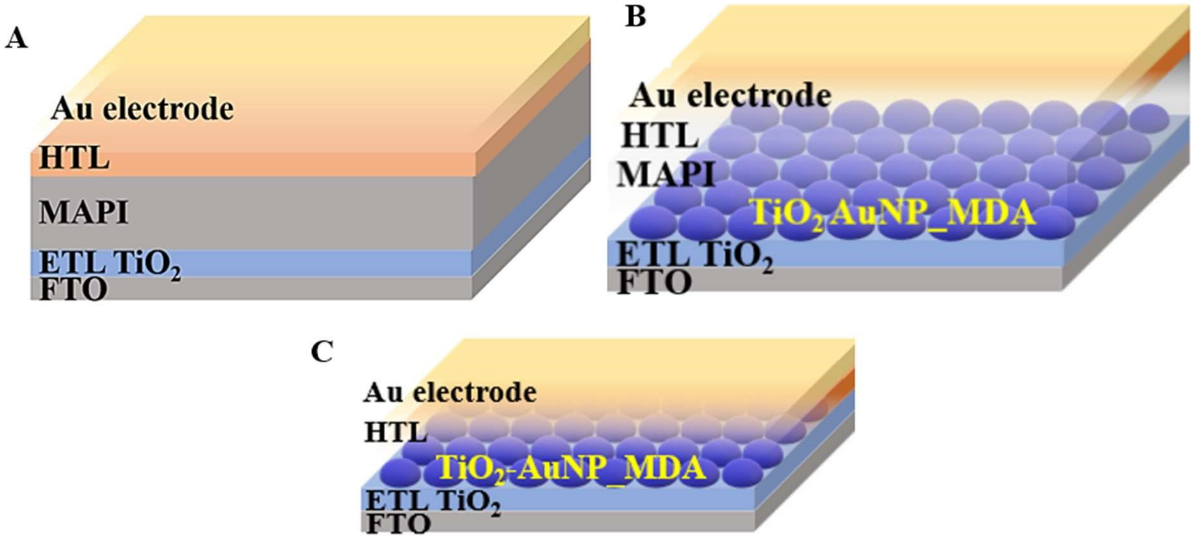

Scheme S1. Scheme of the PSC devices: (A)– standard perovskite PSC with planar architecture, (B) – PSC with TiO_2_ Au_MDA, and (C) – diode-like structure with TiO_2-Au_MDA on the BL/HTL interface.

Contact angle measurements further support the differences in surface wettability caused by the BL treatment. Figure S1A shows the contact angle on the annealed TiO_2 BL using a 7 μL ink droplet, yielding an angle of 56°, indicating a more hydrophobic surface. In contrast, Figure S1B shows a significantly lower contact angle of 16° on the freshly deposited BL, demonstrating its more hydrophilic nature. This difference is attributed to the annealing process, which likely reduces surface hydroxyl groups, increases crystallinity, and modifies surface morphology, all contributing to decreased surface energy. The enhanced hydrophobicity of the annealed BL limits ink spreading, which explains the smaller printed dot diameters observed in subsequent MDA printing.

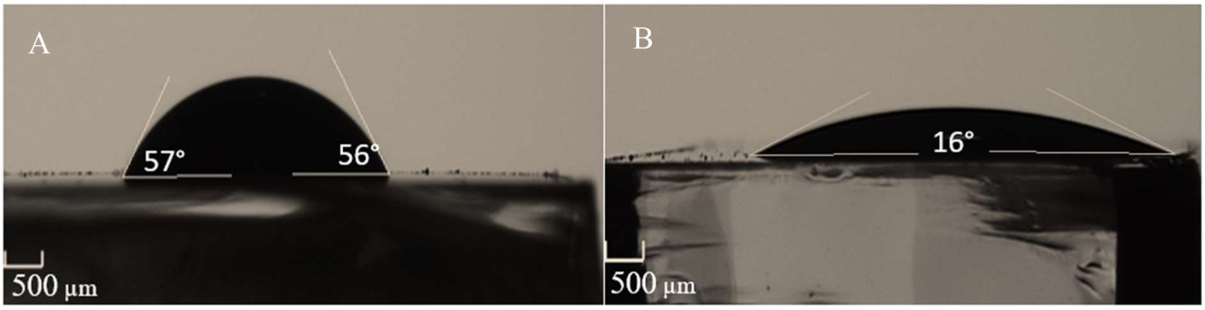

Figure S1. Contact angle measurements of ink droplets on TiO_2 blocking layers (BL) with different surface treatments. (A) Annealed BL shows a contact angle of 56°, indicating increased hydrophobicity. (B) Freshly deposited BL exhibits a contact angle of 16°, indicating a more hydrophilic surface.

9. Characterization

Particle size and zeta potential were measured using a Lightsizer™ 500 instrument (Anton Paar GmbH, Austria). All dispersions were

diluted to a 1:30 ratio with deionized water. Particle size was determined by the dynamic light scattering (DLS) method at 10 °C, with the refractive index set to that of water. The colloidal stability of the nanoparticles was assessed via zeta potential measurements using electrophoretic light scattering (ELS).[4] ELS measurements were performed at 25 °C with a maximum applied voltage of 200 V.

The structural properties of MAPI films deposited on bare glass, glass with printed TiO_2_MDA, and glass with TiO_2-Au_MDA/MAPI layers were analyzed using a SmartLab SE powder X-ray diffractometer (Rigaku, Japan) with Cu-Kα radiation (λ = 1.5460 Å). Measurements were conducted in 2θ geometry (incident angle 3°) over a range of 10–80°, with a step size of 0.03° and scan rate of 0.5°/min. Phase identification was carried out using SmartLab Studio II version. 4.2.44.0 and the Powder XRD module with the ICDD PDF-2 2019 database. Rietveld refinement was applied in the 10–45° range using the Whole Powder Pattern Fitting (WPPF) module. Crystallite sizes were determined using the Comprehensive Analysis module based on the Halder–Wagner method.

Morphological studies of TiO_2-Au_MDA/MAPI films were performed using a TESCAN MAIA3 scanning electron microscope (Czech Republic) operated in both SEM and STEM modes. Detailed compositional and crystallographic analysis of individual TiO_2-Au_MDA structures was carried out via high-resolution transmission electron microscopy (HR-TEM). The size distribution of AuNPs was analyzed from SEM images using ImageJ software with a modified "particle size" plugin.

Optical absorption spectra (recalculated from reflectance data) of the BL/MDA films were acquired using a Sphere Jasco V-750 spectrophotometer (JASCO Co., Japan). Current-voltage (J–V) characteristics of the fabricated PSCs were recorded using a test station (Abet Technologies, USA) equipped with a SunLite™ solar simulator and a Keithley 2400 source meter (Tektronix, UK) under AM 1.5G illumination at 100 mW cm^2, calibrated with a certified silicon reference cell. A stainless-steel mask with a 0.04 cm^2 aperture was used to define the active illumination area of the PSCs.

10. Simulations:

A finite-difference time-domain (FDTD) simulation was employed using Ansys Lumerical to analyze the optical behavior of plasmonic-enhanced perovskite solar cells by solving Maxwell's equations in both spatial and temporal domains. A broadband plane wave source served as the excitation source to probe the local electromagnetic response, while frequency-domain power and field monitors captured spatial field distributions and absorption characteristics. A transmittance monitor was positioned beneath the device stack to quantify the total transmitted power, enabling the calculation of net absorption within the solar cell. The simulated device comprised a multilayer structure: FTO (0.8 μm), TiO_2 (0.06 μm), $CH_3NH_3PbI_3$ (0.39 μm), Spiro-OMeTAD (0.1 μm), and an Au contact (0.12 μm), see Scheme 1, SI. Gold nanoparticles (AuNPs) with

diameters ranging from 10 to 40 nm were embedded within the TiO_2 and $MAPbI_3$ interface to study their plasmonic influence. Nanoparticles were placed at different depths and interfaces of the TiO_2, MAPI, and Spiro layers to evaluate position-dependent effects on optical absorption and short-circuit current density (Jsc). The simulations were conducted under background refractive index conditions (n = 1.4) to mimic encapsulated environments. The dielectric functions of the materials were based on the values reported in the literature,[5,] and a non-uniform automatic mesh ensured accurate spatial resolution.

Results

11. Influence of BL Surface Modifications on MDA Deposition

Figure S2 (**A**) presents SEM micrographs of printed TiO_2-Au_MDA microdot on annealed BL (BL_A), (**B**) higher magnification of the TiO_2-Au_MDA microdot on BL_A. The elemental composition and distribution within the printed TiO_2-Au_MDA microdots were further analyzed using EDS mapping (Figure S2 (**C**)), confirming the presence of gold throughout the microdot regions. The Hirox images of an overview of the dots printed on different types of BL are presented in Figure S2: annealed BL (**BL_A**), freshly deposited BL (**BL_F**), and plasma-treated annealed BL (**BL_P**).

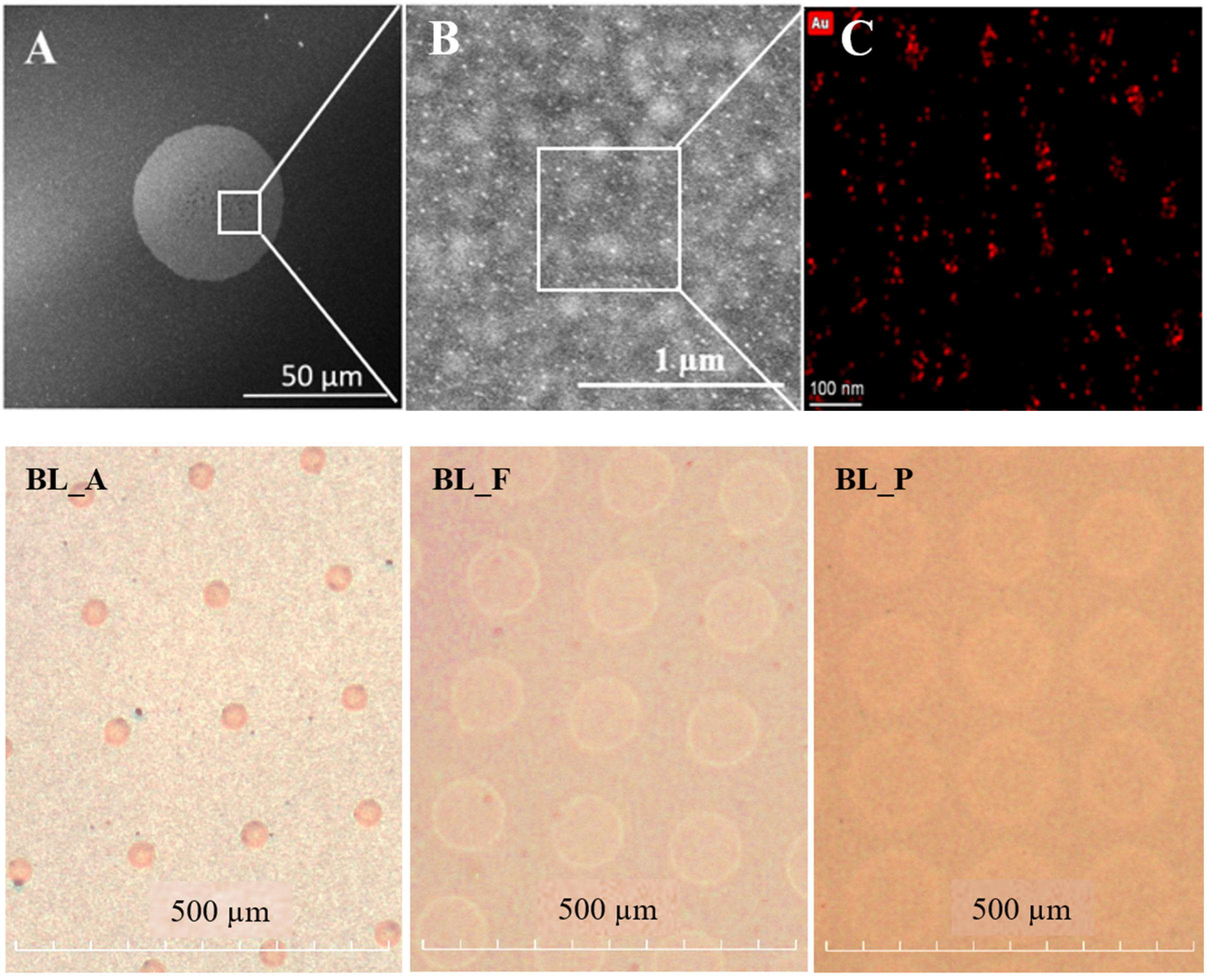

Figure S2 (A) - SEM micrographs showing printed TiO_2-Au_MDA microdot on annealed BL (BL_A), (B) higher magnification of the TiO_2-Au_MDA microdot on BL_A, (C) - EDS mapping results of printed TiO_2-Au_MDA microdot. Hirox images of the overview of the dots printed on FTO and different treatments of BL: annealed BL (BL_A), freshly deposited BL (BL_F), and plasma-treated annealed BL (BL_P).

The box plots in Figure S3 and the best performance in Table S1 present the photovoltaic performance metrics—short-circuit current density (Jsc), open-circuit voltage (Voc), power conversion efficiency (PCE), and fill factor (FF)—for both standard PSCs and those modified with the TiO_2-Au_MDA layer.

Table S1. Photovoltaic performance of the champion devices.

	Diameter of MDA, µm	J, mA/cm^2 ± 0.1	V_{OC}, V ± 0.01	PCE, % ± 0.3	FF, % ± 0.1
Standard		9.1	0.74	2.9	43.3
BL_A TiO_2_MDA	34	13.7	0.75	3.4	32.3
BL_A TiO_2-Au_MDA	35	19.2	0.73	5.2	36.7
BL_F TiO_2_MDA	90	15.8	0.76	4.2	35.1
BL_F TiO_2-Au_MDA	90	16.0	0.69	3.4	30.6
BL_P TiO_2 _MDA	90	11.7	0.69	2.8	34.8
BL_P TiO_2-Au_MDA	90	9.5	0.69	2.2	33.0

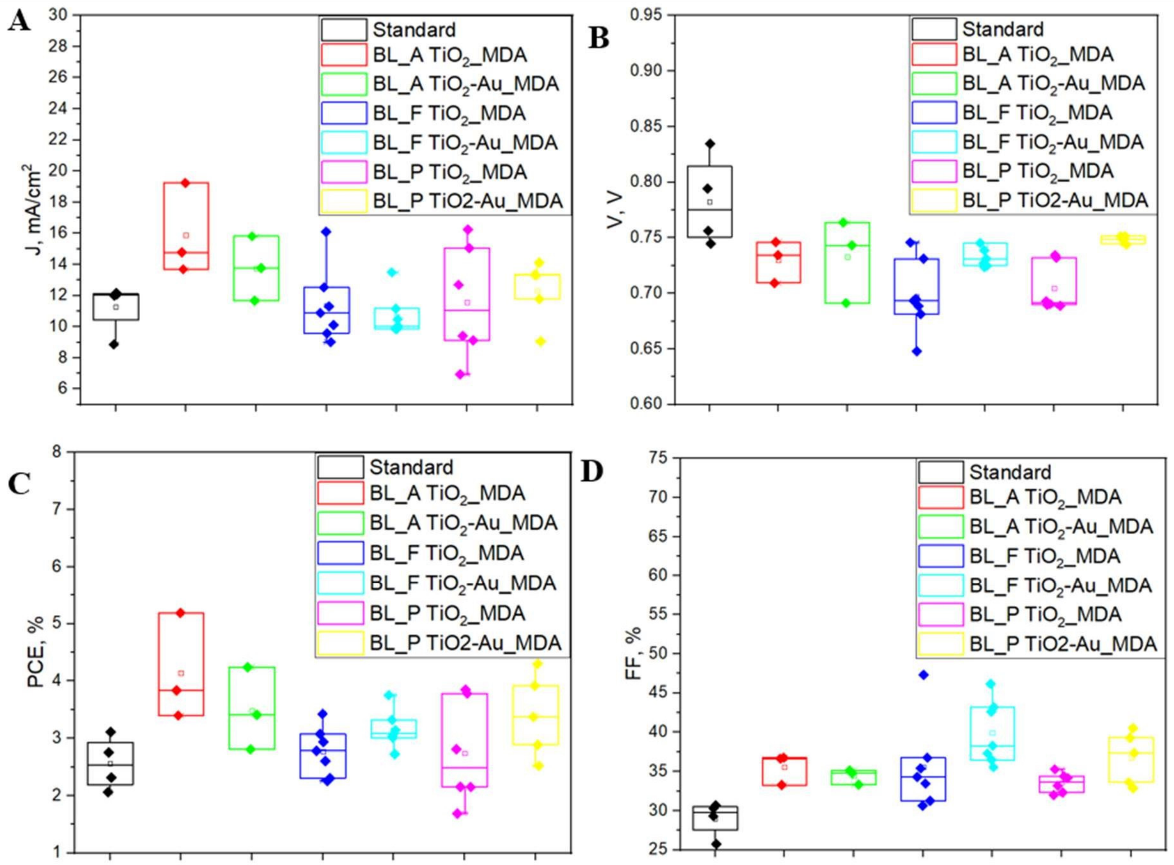

Figure S3. Box plots summarizing the photovoltaic parameters extracted from the J–V curves, including (**A**) short-circuit current density Jsc. (**B**) open-circuit voltage Voc. (**C**) power conversion efficiency PCE. (**D**) fill factor FF. The standard sample presents original PSC without MDAs and TiO_2_MDA samples without AuNP.

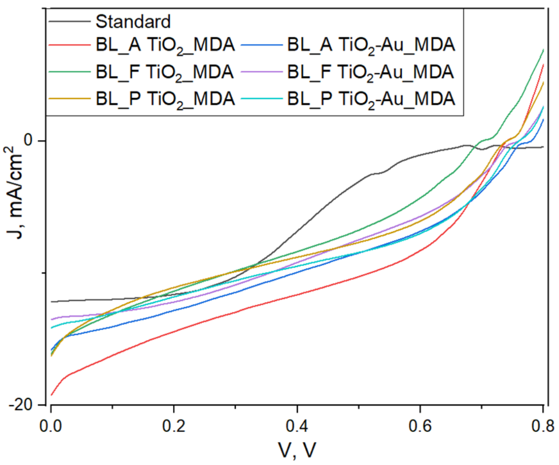

Figure S4 J–V curves of perovskite solar cells (PSCs) with and without TiO_2-Au_MDA modification under and above BL. The curves represent the photovoltaic performance of standard PSCs (black line) and PSCs incorporating the TiO_2-MDA and TiO_2-Au_MDA layer. The standard sample presents original PSC without MDAs and TiO_2_MDA samples without AuNP.

Dots printed above and under BL.

Figure S5 shows the box plot of performance parameters (Jsc, Voc, FF, and PCE) for standard PSCs and TiO_2-modified PSCs with varying AuNP concentrations. The results indicate how gold nanoparticle incorporation affects device performance.

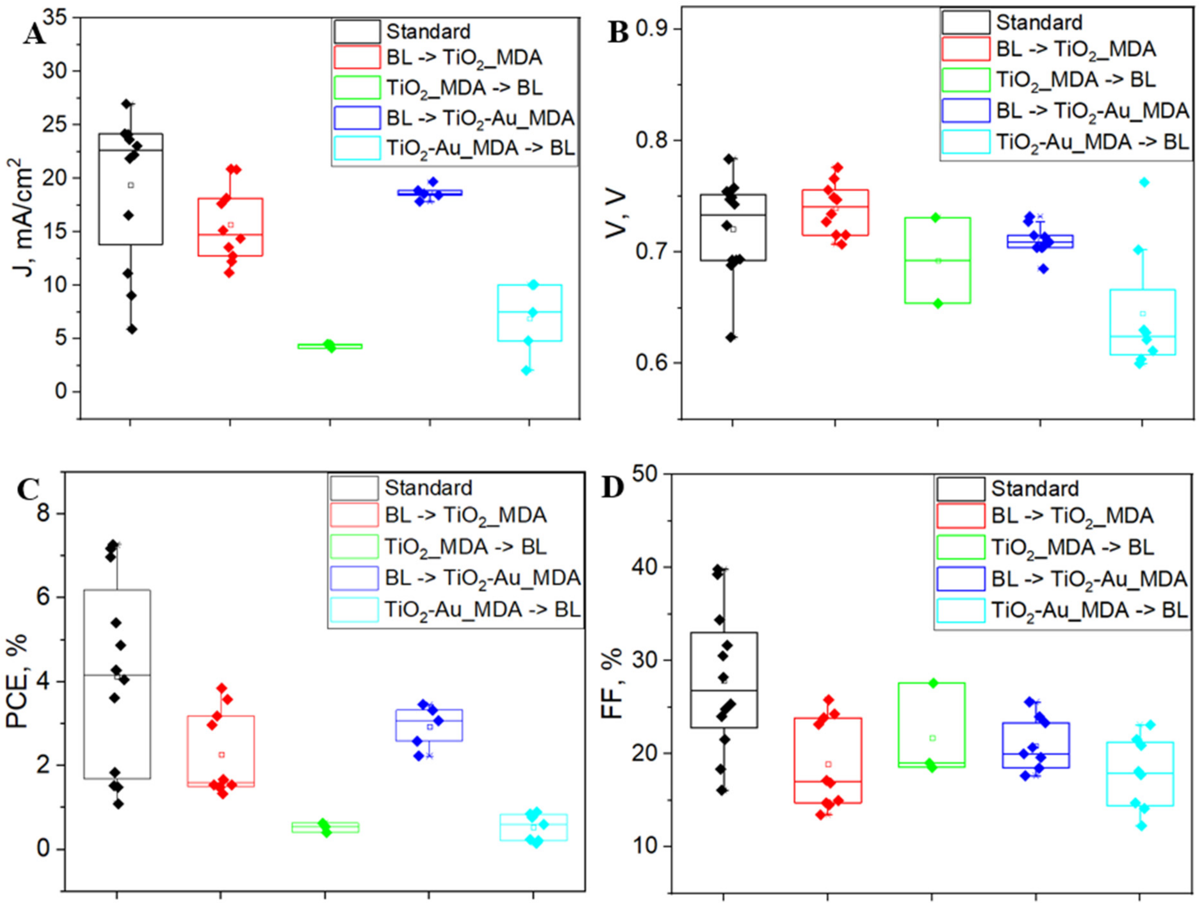

Figure S5 The comparison of PSC performance of the standard sample and with the blocking layer positioned below and under MDAs (**A**) short-circuit current density Jsc. (**B**) open-circuit voltage Voc. (**C**) power conversion efficiency PCE. (**D**) fill factor FF. The standard sample presents original PSC without MDAs and TiO_2_MDA samples without AuNP.

Table S2 shows the performance of all the PSC devices with different architectures. Measurements were conducted on the first day and after seven days of storage in a glove box.

Table S2 Photovoltaic performance of the PCE champion devices measured on day 1 and after 7 days of storage in a glove box.

	Day of measurement	J, mA/cm^2 ± 0.1	V_{OC}, V ± 0.01	FF, % ± 0.1	PCE, % ± 0.3
**Standard**	1	24.2	0.72	40	7.0
	7	26.0	0.78	34	7.2
**BL → TiO_2**	1	26.1	0.69	26	4.7
	7	19.5	0.70	23	3.3
**TiO_2 → BL**	1	5.3	0.73	19	0.6
	7	5.0	0.66	16	0.5
**BL → TiO_2-Au_MDA**	1	18.6	0.73	25.6	3.5
	7	19.0	0.70	23.0	3.3
**TiO_2-Au_MDA → BL**	1	10.0	0.70	20.0	1.0
	7	9.5	0.63	14.0	0.9

Influence of AuNP Concentration on Ink Stability and Photovoltaic Efficiency

Figure S6 MAPI SEM analysis reveals that the morphology of the MAPI films is strongly influenced by the concentration of AuNPs in the underlying TiO_2-xAu_MDA. The pristine MAPI film (on bare glass) appears relatively porous and less uniform, while the sample deposited on the TiO_2-1xAu_MDA substrate exhibits a much more compact and homogeneous surface.

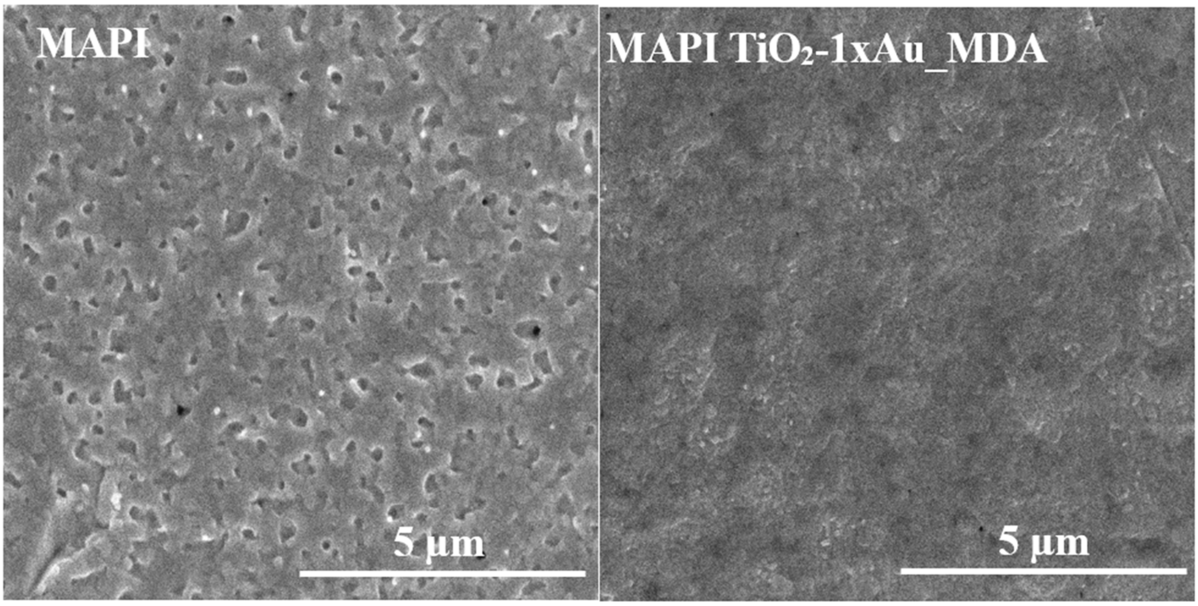

Figure S6 Scanning electron microscopy (SEM) images of MAPI on glass substrates with and without deposited patterned with TiO_2/AuNP MDA. The micrographs illustrate surface morphology and coverage uniformity at Au precursor concentrations (TiO_2-1xAu_MDA).

Figure S7 XRD of the structural characteristics of the MAPI layers deposited on glass substrates with and without inkjet-printed TiO_2-xAu_MDA, prepared with different gold precursor concentrations (1×, 1.5×, and 2×), was determined with an X-ray powder diffractometer (SmartLab SE, Rigaku, Japan) with Cu-Kα radiation (λ = 1.5460 Å). The XRD patterns were registered in 2θ geometry (angle of incidence 3°) in the range of 20–80° with a step of 0.03° and a rate of 0.5°/min. The phase analysis was performed using SmartLab Studio II ver. 4.2.44.0, using the Comprehensive Lattice Parameter Refinement method in the 20–70° range and ICDD (International Centre for Diffraction Data) cards: 01-085-5507; 00-004-0784; 01-071-1166; 00-007-0235 for MAPI, Au, TiO2, and PbI2, respectively. **Figure S7** presents J–V curves of perovskite solar cells incorporating standard PSC and TiO_2 modified with and without AuNPs at varying concentrations (1x, 1.5x, and 2x). The devices were measured under illumination. The Figure highlights the impact of AuNP loading on photovoltaic performance.

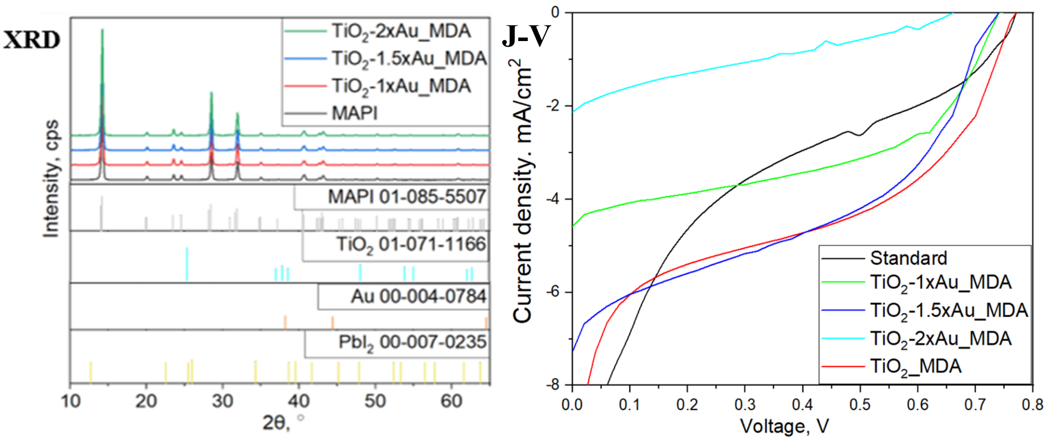

Figure S7 XRD- patterns of full phase analysis with corresponding ICDD (International Centre for Diffraction Data) cards MAPI (grey), Au (orange), TiO_2 (cyan), and PbI_2(yellow). J-V - Current-voltage (J–V) characteristics of perovskite solar cells (PSCs) incorporating different concentrations of AuNPs at the TiO_2–MAPI interface: Standard, TiO_2-1xAu_MDA, TiO_2-1.5xAu_MDA, and TiO_2-2xAu_MDA.

To demonstrate the potential for improved photovoltaic performance, Figure S8 presents devices fabricated using the optimized 1× AuNP MDA configuration, reaching power conversion efficiencies (PCEs) of up to ~8%. These results underscore that, while the current work focuses on the fundamental exploration of plasmonic interface engineering using inkjet-printed TiO_2-AuNP microdot arrays (MDA), the proposed strategy does not inherently limit the absolute device efficiency. Ongoing efforts in our lab are aimed at integrating this approach with advanced perovskite formulations and optimized architectures to further enhance device performance.

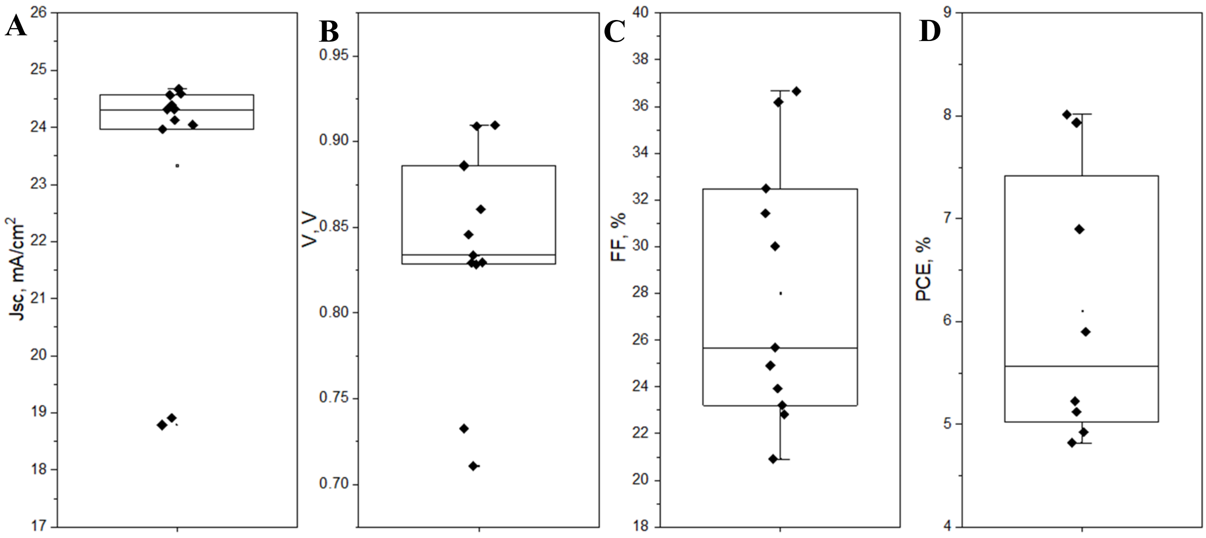

Figure S8 The PSC performance of the modified with 1xAuNP MDA (**A**) short-circuit current density Jsc. (**B**) open-circuit voltage Voc. (**C**) power conversion efficiency PCE. (**D**) fill factor FF.

References

1. Danchuk, V.; Shatalov, N.; Pogreb, R.; Musin, A. Characterization of Sputtered ZnO Blocking Layers with Surface Plasmon Resonance Method. Proceedings of the Seventeenth Russian-Israeli Bi-National Workshop, 2019, pp. 186–193.
2. Keller, K.; Khramenkova, E.V.; Slabov, V.; Musin, A.; Kalashnikov, A.; Vinogradov, A.V.; Pidko, E.A. Inkjet Printing of Sc-Doped TiO_2 with Enhanced Photoactivity. Coatings 2021, 11, 1525.
3. https://doi.org/10.3390/coatings11121525
4. Tomulescu, A.G.; Stancu, V.; Besleaga, C.; Enculescu, M.; Nemnes, G.A.; Florea, M.; Dumitru, V.; Pintilie, L.; Pintilie, I.; Leonat, L. Reticulated Mesoporous TiO_2 Scaffold, Fabricated by Spray-Coating, for Large Area
5. Perovskite Solar Cells. Energy Technol. 2019, 7, 1900922. https://doi.org/10.1002/ente.201900922
6. Brar, K.S.; Verma, M. Measurement of Nanoparticles by Light-Scattering Techniques. TrAC Trends Anal.
7. Chem. 2011, 30, 4–17. https://doi.org/10.1016/j.trac.2010.08.00
8. Ball, J. M., Stranks, S. D., Hörantner, M. T., Hüttner, S., Zhang, W., Crossland, E. J. W., Ramirez, I., Riede, M., Johnston, M. B., Friend, R. H., & Snaith, H. J. (2015). Optical properties and limiting photocurrent of thin film perovskite solar cells. Energy & Environmental Science, 8(2), 602–609.
9. https://doi.org/10.1039/C4EE03224A
